# Collaborating with consumer and community representatives in health and medical research in Australia: results from an evaluation

**DOI:** 10.1186/1478-4505-9-18

**Published:** 2011-05-14

**Authors:** Janet M Payne, Heather A D'Antoine, Kathryn E France, Anne E McKenzie, Nadine Henley, Anne E Bartu, Elizabeth J Elliott, Carol Bower

**Affiliations:** 1 Telethon Institute for Child Health Research, Centre for Child Health Research, The University of Western Australia, Subiaco, Western Australia; 2 School of Population Health, The University of Western Australia, Crawley, Western Australia; 3 Centre for Applied Social Marketing Research, Edith Cowan University, Joondalup, Western Australia; 4 School of Nursing and Midwifery, Curtin Health Innovation Research Institute, Curtin University of Technology, Bentley, Western Australia; 5 Discipline of Paediatrics and Child Health, Sydney Medical School, University of Sydney, Sydney, New South Wales, Australia

**Keywords:** Consumer and community participation, health and medical research, evaluation, Fetal Alcohol Spectrum Disorder, alcohol, impact

## Abstract

**Objective:**

To collaborate with consumer and community representatives in the *Alcohol and Pregnancy Project *from 2006-2008 http://www.ichr.uwa.edu.au/alcoholandpregnancy and evaluate researchers' and consumer and community representatives' perceptions of the process, context and impact of consumer and community participation in the project.

**Methods:**

We formed two reference groups and sought consumer and community representatives' perspectives on all aspects of the project over a three year period. We developed an evaluation framework and asked consumer and community representatives and researchers to complete a self-administered questionnaire at the end of the project.

**Results:**

Fifteen researchers (93.8%) and seven (53.8%) consumer and community representatives completed a questionnaire. Most consumer and community representatives agreed that the process and context measures of their participation had been achieved. Both researchers and consumer and community representatives identified areas for improvement and offered suggestions how these could be improved for future research. Researchers thought consumer and community participation contributed to project outputs and outcomes by enhancing scientific and ethical standards, providing legitimacy and authority, and increasing the project's credibility and participation. They saw it was fundamental to the research process and acknowledged consumer and community representatives for their excellent contribution. Consumer and community representatives were able to directly influence decisions about the research. They thought that consumer and community participation had significant influence on the success of project outputs and outcomes.

**Conclusions:**

Consumer and community participation is an essential component of good research practice and contributed to the *Alcohol and Pregnancy Project *by enhancing research processes, outputs and outcomes, and this participation was valued by community and consumer representatives and researchers. The National Health and Medical Research Council in Australia expects researchers to work in partnership and involve consumer and community representatives in health and medical research, and to evaluate community and consumer participation. It is important to demonstrate whether consumer and community participation makes a difference to health and medical research.

## Background

The World Health Organization stated in 1978 in the Declaration of Alma-Ata that "people have the right and duty to participate individually and collectively in the planning and implementation of their health care [1]". The declaration contributed to the tenets of consumer participation [2-4] in health care, health services and health and medical research in the United Kingdom [5], western European countries [6], North America [7,8] and Australia [9]. In health and medical research, consumer participation is considered morally [10-12] and ethically correct practice [2]. It supports the advancement of accountable, open and democratic [2,4,13-15] involvement of citizens in publicly funded research [10,12,14].

Consumers are reported to make an important contribution to the quality of health and medical research [2,4,10,13,16] through their experiential expertise that complements the expertise of researchers [12,15], by providing unique [2,11] and broader community perspectives [10,15,17], and drawing attention to issues of which researchers may not be aware [11]. In the practical undertaking of health and medical research, consumers may contribute to all aspects of research including recruitment of research participants [16,18], improving information that is provided to participants [18], aiding the dissemination and implementation of results [4,10,11,16], improving the uptake of research findings [4,19], providing legitimacy for research [18], and encouraging greater understanding of research in the community [12]. Consumer participation in health and medical research is often a policy directive [2] and a requirement of funding organisations [20] but should not only be practised for these reasons. Rather, it is an essential component of good research practice [11,16]. However, there is little evidence of the impact of consumer involvement in health and medical research [12,18,21,22], or of instruments and frameworks for its measurement [23]. A need for development of methods of evaluation has been identified [2,4,24,25] and an opportunity exists to develop robust and rigorous methods for evaluation of consumer participation in health and medical research [18].

In Australia, the National Health and Medical Research Council (NHMRC) has stated "To ensure the integrity of health and medical research and accountability to the community, a researcher or research organisation must be able to fully justify any decision to proceed *without *wider participation of consumers" [9]. The NHMRC is the statutory body that advises the community and Government on standards of individual and public health and funding of medical and public health research and training, and develops guidelines and standards for the ethical conduct of health and medical research [9]. In 2001, it co-endorsed the *Statement of Consumer and Community Participation in Health and Medical Research *[9] with the Consumers' Health Forum, Australia's peak non-government organisation representing consumers on national health care issues. In 2005 the NHMRC published *A Model Framework for Consumer and Community Participation in Health and Medical Research *[26] and a *Resource Pack for Consumer and Community Participation in Health and Medical Research *[27] that provided further guidance to developing consumer and community participation (CCP) in research.

The NHMRC recognised the potential contribution of consumers and their right to be involved in health and medical research as equal partners in the development of research goals, strategies, methodologies and dissemination of research that were "open to informed public scrutiny and debate" and ensured "the integrity of research and accountability to the community for the quality of the research" [9]. The Statement declared that consumer participation was the responsibility of both researchers and consumers who were to be respectful of each others' knowledge and "ensure that ethical requirements are met, that there is value to the research" and that "consumers and researchers share a vision of working in partnership based on understanding, respect and shared commitment to research that will improve the health of humankind" [9].

Health consumers have been defined as patients, carers, service users, users, lay persons, clients, and citizens [2,22,28-30]. In the context of research, the NHMRC defined consumers not as participants in a research study [26] but as health consumers, consumer representatives and community members [9,26]. The NHMRC's description of consumers' levels of participation were derived from Bastian [28] and explained in terms of "none, manipulation, restricted scope, open involvement and wide participation". However, different levels of consumer participation have been described [2,16,22,28,31,32].

Recommendations in 1998 by the Health and Medical Research Strategic Review (the Wills' Report) [33] that the community should be involved in the research effort, and the awarding of a NHMRC Capacity Building Grant in 2002 enabled the appointment of a consumer research liaison officer jointly at the Telethon Institute for Child Health Research (the Institute) and School of Population Health at The University of Western Australia. In 2004 these two organisations commenced a program to enhance and increase consumer participation in their health and medical research that included the following components: employing a dedicated consumer advocate (appointed in 2004); having a well developed governance and policy framework for participation that included Consumer and Community Advisory Councils at each organisation (endorsed and convened in 2004 [34,35]); budgeting in research grant applications for participation activities; developing a range of best-practice models for consumer and community participation; conducting training events relevant to participation for researchers and consumer and community representatives (CCRs); and arranging forums to bring researchers, other professions and consumer and community members together. In 2007, a book based on research involving consumers at the two institutions, titled *Consumer and Community Participation in Health and Medical Research: A Practical Guide for Health and Medical Research Organisations *was published [32]. It outlined ingredients for success in community and consumer participation at an organisational level and in individual research projects, using researchers' experiences and stories from consumers about their involvement in research.

One of the principles included in the Institute's Consumer and Community Participation Policy states that "Consumer and community participation, and the Institute's support for participation will be evaluated in consultation with consumers, community members and researchers, and made available to the public" [35]. This is in line with the NHMRC, which noted that "consumer participation in research should also cover evaluation of the participation in terms of positive social and medical change" [9].

Before we commenced the *Alcohol and Pregnancy Project *in 2006, we committed to collaborating with CCRs in the research, not as a condition of funding, but to comply with the NHMRC statement on consumer participation in research [9], and to conduct good research practice [11,16,36]. We anticipated that CCP would add value to the research. The project involved synthesising published material; conducting formative research with health professionals [37] and Aboriginal and non-Aboriginal women of childbearing age; developing educational resources about the prevention of prenatal alcohol exposure and Fetal Alcohol Spectrum Disorder; distributing the resources to over 3,000 health professionals in Western Australia (Aboriginal health workers, allied health professionals, community nurses, general practitioners, obstetricians, and paediatricians); and surveying these health professionals to evaluate the project. At the end of the project we conducted a further evaluation to answer the question: what were researchers' and CCRs' perceptions of the process, context and impact of CCP in the project. In this paper we describe how we collaborated with CCRs and report the results of this evaluation.

## Methods

### Establishing the Steering Committee for the *Alcohol and Pregnancy Project*

A Steering Committee was responsible for research and resource decisions essential to achieving the outputs and outcomes of the *Alcohol and Pregnancy Project*. This committee comprised 20 interdisciplinary researchers: 16 investigators, including the consumer research liaison officer and the project manager; three project team members and a representative of an Aboriginal research network (three researchers did not continue to the end of the project). This was the first time that the researchers involved in the project had collaborated formally with CCRs in research.

### Establishing consumer and community representation

We established two consumer and community reference groups to provide a forum for participation and consultation, and convey community perspectives and guidance to enhance successful delivery of the project. The consumer research liaison officer and researchers who were also members of the Aboriginal community nominated the CCRs and advised us about setting up the groups. According to their advice we set up an Aboriginal Community Reference Group which only comprised CCRs who were members of the Aboriginal community because of the sensitivity of the topic of alcohol consumption in pregnancy and the different issues faced by Aboriginal and non-Aboriginal women. They also advised us to omit 'consumer' from the name of the Aboriginal Community Reference Group as other Aboriginal research projects had previously used the term 'community refererence group' [38], and to include a male member in the Aboriginal Community Reference Group as the topic of alcohol and pregnancy was of concern to both women and men is the Aboriginal community. We also set up a Consumer and Community Reference Group comprising non-Aboriginal members of the community. Both groups had three CCRs who were women of childbearing age because such women would be the recipients of health professionals' advice about alcohol consumption in pregnancy.

We developed Terms of Reference using the NHMRC Statement [9], Model Framework [26], and Resource Pack [27], the Institute's Consumer and Community Participation Policy [35] and guidance from the consumer research liaison officer. The Terms of Reference (Appendix 1) comprised six statements about the level of CCP [2,16,22,28,31,32] with an additional one for the Aboriginal Community Reference Group pertaining to the relationship between the project and cultural, spiritual and social values of Aboriginal people, with respect for the richness and integrity of their cultural inheritance [39] (Table 1).

**Table 1:** CCRs' agreement with statements about CCP in the *Alcohol and Pregnancy Project*

**Compliance with the project Terms of Reference - to provide comment and advice on**:	**Agreement**^**a **^**with statement** (n = 7)		
	Agree	Unsure	Disagree
1. The relationship between the project, cultural, spiritual and social values with respect for the richness and integrity of the cultural inheritance of Aboriginal people^b^	1SA, 2A		
2. The management of data, publications, and the protection of individual and/or community identities during the project ^c^	1SA, 4A	1U	
3. The methodology, conduct, dissemination of results and potential outcomes of the project^c^	1SA, 5A		
4. Alcohol use in pregnancy and how it was dealt with in a culturally sensitive way ^c^	2SA, 4A		
5. Project documents such as consent forms and participant information sheets, interview guides, questionnaires and health promotion resources	3SA, 4A		
6. The final report and draft reports and manuscripts before they were published in scientific journals^c^	2SA,1A	3U	
7. The preparation and development of summaries for the community and dissemination^c^	2SA, 3A		1D
**Compliance with Telfords' principles of successful consumer involvement:**[46]			
1. Roles of researchers were agreed between researchers and consumers	6A	1U	
2. Researchers budgeted appropriately for the costs of consumer and community representatives	2SA, 2A	3U	
3. Researchers respected the differing skills, knowledge and experience of consumer and community representatives	4SA, 3A		
4. Consumer and community representatives were offered training and personal support to enable them to be involved in the research	1SA, 3A	3U	
5. Researchers had the necessary skills to involve consumer and community representatives in the research process	1SA, 5A	1U	
6. Consumer and community representatives were involved in decisions about how participants were both recruited and kept informed about the progress of the research	1SA, 5A	1U	
7. Consumer and community representatives' involvement was described in research reports	1SA, 4A	2U	
8. The research findings were available to consumer and community representatives, in formats and language that could be easily understood	1SA, 5A		1D
**CCRs' perceptions of their participation in the project**:			
1. Consumer and community representatives' viewpoints were valued by the researchers	5SA, 2A		
2. Consumer and community representatives' contributions were recognised by the researchers	3SA, 4A		
3. Consumer and community representatives' participation added value to the project	5SA, 2A		

^a ^Possible options: Strongly agree (SA), agree (A), unsure (U), disagree (D), strongly disagree

^b ^This Term of Reference was specifically for the Aboriginal Community Reference Group (n = 3)

^c ^Respondent stated the question was not applicable

### Consumer and community participation in the project

The two groups met separately 12 times during the three year project. Before participating in 2006, CCRs were given verbal information and documents about the project by researchers: Information for the Community (Appendix 2), Project Summary (Appendix 3), and Terms of Reference (Appendix 1). In 2008 they participated in training workshops conducted by the consumer research liaison officer. Meetings were attended by three researchers, including the project manager and another researcher who chaired the meetings. An agenda and notes from the previous meeting and supporting documents were circulated, the Terms of Reference were reviewed at the beginning of each meeting and notes were recorded. Reports on these meetings were provided by the project manager to the Steering Committee. CCRs were paid sitting fees to acknowledge their contribution of time and out of pocket expenses. Between meetings, they provided comments on documents that were circulated by email. As a result of a recommendation of the project manager, CCRs became part of the Steering Committee in the final year of the project.

### Consumer and community representatives' contribution to the project

In accordance with the Terms of Reference, CCRs offered their perspectives on all aspects of the project including attitudes of women toward alcohol use in pregnancy and women's expectations about information and advice provided by health professionals about alcohol and pregnancy. CCRs also contributed their perspectives and comments on:

(1) the development of documents during the formative research, including consent forms, information sheets, topic guides for focus groups and in-depth interviews;

(2) the educational resources for health professionals (38 page booklet, a double-sided laminated fact sheet, a wallet card for health professionals to give to women, and a desk-top calendar with each monthly view displaying the message *No Alcohol in Pregnancy is the Safest Choice*);

(3) a questionnaire that was sent to health professionals to evaluate the project;

(4) research dissemination activities including abstracts submitted for presenting at conferences, PowerPoint presentations, recommendations for the final report, recommendations to encourage sustainability of the resources, and articles prepared for publication in peer reviewed journals; and

(5) proposals for future research developed during the three year project and involving research design and planning, proposing research questions, questionnaire development, data collection and grant applications.

### Evaluating consumer and community participation

A search of the literature did not reveal a suitable form of evaluation of CCP in health and medical research. Various approaches to evaluation [2,3,13,16,22,30,40-46] were then investigated and because a validated instrument was not available, we developed a questionnaire to assess the process, context, and impact of CCP in the project. The two page self-administered questionnaire for CCRs comprised 22 questions (including an additional question for the Aboriginal Community Reference Group) with both open and closed response options (strongly agree, agree, unsure, disagree, strongly disagree, not applicable).

To address the process we assessed compliance with the Terms of Reference that were agreed by CCRs and researchers at the commencement of the project. We sought agreement to six (seven for Aboriginal CCRs) statements with closed response options (Table 1). To address the context we assessed Telford's [46] principles of successful involvement in National Health Service research (Table 1) by seeking agreement with eight closed response options, and also three closed response options about participation in the project (Table 1). Assessment of the impact was based on Hanley's [16] briefing notes for researchers on evaluation. Four open-ended questions addressed what difference CCP had made to the project, what did and did not work well, and what changes should be made for future projects (Table 2).

**Table 2:** Open-ended questions asked in the evaluation of researchers' and CCRs' perceptions of the impact of CCP in the *Alcohol and Pregnancy Project*

Open-ended questions	Researchers n = 15 (%)		CCRs n = 7 (%)	
	Responded	Did not respond	Responded	Did not respond
What difference do you think consumer and community participation make to the project?	14 (93.3)	1 (6.7)	6 (85.7)	1 (14.3)
What was learned about consumer and community participation that worked well?	11 (73.3)	4 (26.7)	6 (85.7)	1 (14.3)
What was learned about consumer and community participation that did not work well?	11 (73.3)	4 (26.7)	5 (71.4)	2 (28.6)
What changes in consumer and community participation can you suggest for future projects?	13 (86.7)	2 (13.3)	6 (85.7)	1 (14.3)

The questionnaire and a copy of the Terms of Reference, a Project Summary and notes from the meetings were sent to all the CCRs who had participated in the project (n = 13) including those who attended as a proxy (n = 4). In addition, the 16 researchers remaining in the project, excluding the project manager who analysed the data, were asked to complete the four open-ended questions. The questionnaires were returned to a person who was not a researcher and de-identified so the analysis could be conducted without awareness of the identity of the respondent.

Responses from the closed response options were tabulated (Table 1). Responses to CCRs' and researchers' open-ended questions were transcribed verbatim. Two authors (JP and KF) independently developed a coding guide, and together used a constant comparative method [47] of qualitative analysis to generate, contrast and define the categories.

Approval for the *Alcohol and Pregnancy Project *was obtained from the Women's and Children's Health Services Ethics Committee, the Western Australian Aboriginal Health Information Ethics Committee, and the Edith Cowan University Human Research Ethics Committee.

## Results

Fifteen questionnaires (93.8% response) were completed and returned from 16 researchers. Of the questionnaires sent to eight members of the Aboriginal Community Reference Group (four of whom had attended as a proxy) and to five members of the Consumer and Community Reference Group, seven were returned completed (53.8% response), three from members of the Aboriginal Community Reference Group and four from members of the Consumer and Community Reference Group.

Regarding the process and context of CCP, the majority of CCRs agreed that the research complied with the project Terms of Reference and with Telford's [46] eight principles of successful consumer involvement in research. The majority of CCRs also agreed that their participation had added value to the project and that researchers respected and valued their differing skills, knowledge and experience (Table 1). Key findings from the qualitative analysis of the four open-ended questions are addressed below. Quotes have been selected for the way in which they encapsulated the category and could be incorporated into its description. Each participant has a unique identifier (R1-15 for researchers and CCR1-7 for CCRs) in order to indicate the range of views expressed.

### What difference did consumer and community participation make to the project?

#### Scientific and ethical standards are enhanced through allowing "hidden voices"

Researchers thought "the scientific and ethical standards of the project (were) enhanced" [R5], and "that (CCP) likely increased credibility of the project and participation" [R12]. They acknowledged a "good rounding of consumer input with scientific/research fact" [R3] and that CCP was "fundamental" [R15] to the research process. Both researchers and CCRs appreciated the contribution of "different perspective(s)" [CCR5] to the project by the CCRs and the feedback that was provided, in particular, with regard to the "development of project resources" [the educational resources for health professionals] [R2]. Researchers pointed out that "allowing hidden voices equal input validates and enriches both data collected and outcomes" [R14] and that CCRs' and researchers' "collective perspective empowers the study's wholesomeness" [R13]. They thought "it was good to hear the voice of real consumers" [R6].

CCRs also spoke of the value their involvement contributed to the quality of the research, particularly in terms of "ensur(ing) that the project researchers kept the end consumer in mind through all stages" [CCR1]. They thought they offered "real life examples" [CCR3], specifically by taking into account "cultural sensitivities" [CCR4], "other demographics" [CCR6] and a variety of situations and target groups that were appropriate for the research.

#### Gave the project legitimacy and authority

CCRs believed "the involvement of the community had (a) significant influence on the success of the project. It provided the project legitimacy and authority" [CCR7]. They thought that obtaining realistic viewpoints from CCRs made the project "more meaningful" [CCR6] although this required time and effort. "While consumer participation is time consuming and not core to the research project this has been an example of how if the time and effort is put in a rewarding outcome is the result" [CCR1].

#### "Seek direction when tackling difficult issues"

Researchers appreciated being able to seek direction when tackling difficult issues such as Fetal Alcohol Spectrum Disorder and directly approaching the community with a message about the prevention of alcohol consumption during pregnancy. They noted it was "very important to have the input from consumers especially for projects such as this where we are going directly to the public with our messages" [R1] and that it "assists when people raised concerns of stigmatisation or contention" [R13]. CCRs' participation in decision making processes was "reassuring" [R10] for researchers and confirmed that "the research team was not working in isolation" [CCR7]. Researchers were left with positive feelings about CCP. "Leaves behind good feelings about research and anticipation for the next one" [R14].

### What was learned about consumer and community participation that worked well?

#### The high value placed on consumer and community participation

CCRs referred to the importance of the "high value placed on consumer participation" [CCR1]. They felt valued, listened to, and were "very supported" [CCR1] by the researchers. "The feeling was one of acceptance by all persons present at each meeting" [CCR6]. They perceived a "genuine desire for our participation and contributions" [CCR1] and "a very warm grateful attitude on behalf of the researchers towards we consumer representatives" [CCR2]. CCRs felt that they were an "important part of the project" [CCR2], and that they influenced decisions. "The team ensured that representatives always felt valued and that this wasn't a token activity to satisfy the project requirement" [CCR1].

#### Respect for opinions and expertise

Researchers thought that CCP worked well in research. They mentioned that "they should always be there" [R13] as part of research and that "it DOES work well" [R1]. Researchers noted the importance of respecting the "opinions and expertise" [R7] of CCRs and also of understanding that people's "different situations.... can affect their ability to contribute" [R11] and that "opinions may differ considerably depending on experience" [R6].

#### Getting advice from people not looking at the project through a research lens

Researchers were able to obtain advice from CCRs and thought "it was important to get advice from people who were not looking at the project through a research lens" [R10] and to "learn directly from them" [R1]. They appreciated CCRs input into specific areas of research and reciprocated by keeping CCRs informed about the project and acknowledging their comments. CCRs appreciated this and felt "it was instructive in that the community had greater involvement in the research process which had the effect of demystifying and providing another view of research and researchers" [CCR7].

### What was learned about community and consumer participation that did not work well?

#### Structure of the meetings

CCRs commented on the time period of three months between the reference group meetings and the lack of continuity that made it difficult to remember details from the previous meeting, "...because the gap between meetings meant I forgot some of what I was told before" [CCR1]. CCRs also noted that there was a "high learning curve" [CCR1] and it was "difficult to initially feel comfortable about the subject matter and the research process" [CCR1]. A contrasting view from a CCR was that "I could have been given more executive tasks to do" [CCR2]. Researchers also supported more frequent meetings of the reference groups as "often the reporting to the consumer reference groups was on a retrospective basis and did not facilitate the involvement of the consumer reference groups in key decisions" [CCR5].

### What changes in consumer and community participation can you suggest for future projects?

#### Build on the good work

Researchers and CCRs endorsed CCP in research. Researchers thought it was important to "retain the focus on community input" [R14], to "include (CCRs) earlier in everything" and that "any way to increase their participation is always good" [R2]. CCRs offered guidance to researchers to include them earlier in future projects and to continue to involve CCP in research. "This process has achieved a lot in the relationships between researchers and the community. I suggest that they keep building on this good work" [CCP7].

#### Structure of the meetings

Both CCRs and researchers offered advice about conducting meetings for future research. Researchers suggested increasing the number of CCRs and to "allow adequate time to update and brief new and recently absent consumer and community reference group members on project objectives and current activities" [R5]. CCRs proposed holding the initial few meetings closer together and to "present in PowerPoint form a very dumbed down explanation of the project and the problem its trying to solve" [CCR1]. Another view that was generally agreed by CCRs and researchers for future projects was that CCRs should become part of the Steering Committee. "When the group was put in as part of the steering committee this indicated the importance of consumer contributions" [CCR1] and "consumer and community members could be part of the team rather than a separate group" [R11].

#### "Enhance engagement with the project"

In future projects, researchers wanted to enhance CCRs' engagement with the project so they "can see the difference they are making to the project" [R5]. It was suggested that this could be achieved through developing a formalised structure for feedback and informing CCRs regularly how their involvement and suggestions were incorporated in the project. CCRs offered guidance to researchers to "ask consumers what are their opinions on specific items frequently" [CCP4], advising against the use of jargon and acronyms and suggesting workshops to disseminate information to the community.

## Discussion

We have described our partnership with CCRs in health and medical research and an evaluation that addressed the process, context and impact of CCP. CCRs and researchers thought CCP made a difference to the process, outputs and outcomes of this research. CCRs agreed that the research complied with the project Terms of Reference and Telford's [46] principles of successful consumer involvement. Both consumers and researchers agreed that CCP made a valuable contribution to the research and also identified areas for improvement and offered suggestions for future research.

Researchers thought CCP contributed to project outcomes by enhancing scientific and ethical standards, providing legitimacy and authority, and increasing the project's credibility and participation. They saw it was fundamental to the research process and acknowledged CCRs for their excellent contribution. CCRs thought that CCP had significant influence on the success of project outcomes and were able to directly influence decisions about the research. Researchers acknowledged their respect for CCRs' opinions and expertise, and that the process and outcomes of CCP in research worked well. Both researchers and CCRs thought CCP was essential for the development of the major output of the project, the educational resources for health professionals about prevention of prenatal alcohol exposure and Fetal Alcohol Spectrum Disorder. However, researchers and CCRs reported some structural limitations of the reference group meetings and also made positive suggestions how these could be improved for future projects. Notwithstanding this, they both strongly supported CCP and the earlier inclusion of CCRs in future research.

We believe that these qualitative and quantitative data show that CCRs and researchers achieved the NHMRC requirements [9] for CCP in health and medical research to: (1) open the project to public scrutiny - by getting advice from people not looking at the project through a research lens; (2) involve CCRs as equal partners in the research - by placing a high value on CCP, and agreeing and complying with the project Terms of Reference; (3) be respectful of each others' knowledge - by respecting CCRs' opinions, knowledge and expertise; (4) ensure ethical requirements are met - by enhancing ethical standards through allowing "hidden voices"; (5) ensure integrity and accountability to the community for the quality of the research - by giving the project legitimacy and authority; and (6) add value to the research - by CCRs commenting and advising on all aspects of the project and researchers seeking direction when tackling difficult issues.

It may appear that we had a poor response from CCRs to the questionnaire because only seven of 13 questionnaires distributed were completed and returned. To be inclusive, we sent the questionnaire to all CCRs who had ever attended a meeting, and in so doing, considered that CCRs who had attended only one or very few meetings as a proxy may not respond. This would reduce the response rate, although we are not certain whether this occurred as the data were de-identified before reporting. Although the numbers involved in this evaluation are small, we believe the results are informative and worthy of reporting. It is likely that future evaluations from similar research projects may also be based on small numbers. It is important to build a culture of continuous quality improvement [48]; to evaluate whether CCP makes a difference and improves the process, outputs and outcomes of research; to apply the lessons learned in future projects; and make this information available to other researchers and CCRs.

Our research partnership with CCRs required additional use of research resources and personnel including time for researchers to plan, coordinate and manage processes involving communication, meeting procedures and provision of documentation. A sufficient budget is also needed for sitting fees and refreshments, note taking, venue and parking. We were fortunate to have administrative support for meetings and note taking, a venue for meetings and parking provided by the Institute at no additional cost. It is important to allow for these resource costs when planning CCP so that the research is high quality and achieved within the project time frame and budget. We believe our research partnership with CCRs benefited by being conducted at an organisation that enabled participation through its Consumer and Community Advisory Council and consumer research liaison officer. Because there was leadership and commitment at an organisational level we received valuable support from the consumer research liaison officer when developing our proposal for participation and throughout the project. A national survey of research funders and research organisations conducted in Australia in 2008-2009 confirmed that it is important to have a person within a research organisation who has skills and commitment to facilitating CCP [36], but disappointingly, reported that only 43% of research organisations had policies and/or protocols requiring consumer involvement [36].

Although the NHMRC asserts that CCP should be evaluated in terms of "positive social and medical change" [9] there is limited guidance on how to do this and we did not locate a suitable form of evaluation in the literature. In the context of this project we were not able to conduct a randomised controlled trial to provide a high level of evidence and realise that these results may not be generalisable to other research. Nonetheless, a strength of our evaluation is that much of it was based on methods published by leading authors on CCP [16]. It has been proposed that the right conditions (the context) and explicit conduct (the process) need to be in place for CCP to have beneficial impact in health and medical research [23]. This is supported by the results of our evaluation. In future projects, we would conduct a similar evaluation on a yearly basis of the process, context and impact of CCP with researchers and CCRs and make changes if they were required [49]. In addition, we would record how CCRs' involvement and perspectives were incorporated in the project, and if they were not, the reasons why. Researchers and CCRs could then see where CCP had made a difference to the project.

## Conclusion

We have provided practical guidance on how we collaborated with CCRs in a health and medical research project. We designed and conducted an evaluation to answer the research question 'what were researchers' and CCRs' perceptions of the process, context and impact of CCP in the project'. Our evaluation framework may add to the limited evidence [2,4,18,24,25] of methods of evaluation and contribute to the development of robust, rigorous and validated methods of evaluation of CCP in health and medical research. By describing our experience and presenting researchers' and CCRs' perceptions of the process, context and impact of CCP in the *Alcohol and Pregnancy Project *we hope to encourage other health and medical research organisations to become involved with consumers and the community in research [36]. Finally, we conclude that CCP in health and medical research is an essential component of good research practice [11,16] and contributed to the *Alcohol and Pregnancy Project http://www.ichr.uwa.edu.au/alcoholandpregnancy*by enhancing research processes, outputs and outcomes, and that this participation was valued by CCRs and researchers. The NHMRC in Australia expects researchers to work in partnership and involve CCRs in health and medical research, and to evaluate CCP. It is important to demonstrate whether CCP makes a difference to health and medical research.

## Declaration of competing interests

The authors declare that they have no competing interests.

## Authors' contributions

JP, NH, HD'A, AB, EE and CB originated the project. All authors contributed to conceptualising ideas, interpreting findings, and reviewing drafts of the article. JP supervised all aspects of project implementation and analysis, led the writing and completed the first draft. KF contributed to implementation, coordination of the project and analysis. AMcK contributed to consumer and community participation. All authors read and approved the final manuscript.

## Appendix 1: Alcohol and Pregnancy Project: Terms of Reference for the Consumer and Community Reference Group

### 1. The Role of the Consumer and Community Reference Group

The role of the Consumer and Community Reference Group is to provide a consumer/community perspective on all activities of the *Alcohol and Pregnancy Project*. Because of the cultural sensitivity of the topic of alcohol consumption in pregnancy and the different issues for Aboriginal and non-Aboriginal women there will be an Aboriginal Community Reference Group for Aboriginal women and a Consumer and Community Reference Group for non-Aboriginal women.

### 2. Accountability

The Consumer and Community Reference Group will report to the *Alcohol and Pregnancy Project *Steering Committee through the Project Manager.

### 3. Membership

Membership of the Consumer and Community Reference Group will comprise:

• Three members of the general community who are not project investigators

Research Support for the Consumer and Community Reference Group:

• Ms Heather D'Antoine, Chairperson, Telethon Institute for Child Health Research, WA;

• Ms Anne McKenzie, Telethon Institute for Child Health Research, WA;

• Ms Jan Payne, Project Manager, Telethon Institute for Child Health Research, WA; and

• Ms Helen Daley, Administrative Secretary, Telethon Institute for Child Health Research, WA.

#### 3.1 Meetings

The Consumer and Community Reference Group shall meet on dates to be decided on the first Wednesday of the month, or at other agreed times, not less than four times per year.

#### 3.2. Quorum

A minimum of two Consumer and Community Reference Group members and two Research Support members (one of whom is Aboriginal) is required for the meeting to be recognised as an authorised meeting.

#### 3.3. Payment

An honorarium will be paid to Members of the Consumer and Community Reference Group (excluding members who provide Research Support) to acknowledge their contribution of time and out of pocket expenses.

#### 3.4 Method of Appointment

The Steering Committee shall appoint three community members to the Consumer and Community Reference Group following advice from the Health Consumers' Council in Western Australia.

#### 3.5 Term of Appointment

Members of the Consumer and Community Reference Group will be appointed for a two year term. Members may be re-appointed at the end of the term.

#### 3.6 Chairperson

The Chairperson, Ms Heather D'Antoine shall convene the Consumer and Community Reference Group meetings. If she is not available she will nominate a person responsible for convening and conducting that meeting.

#### 3.7 Agenda Items

All agenda items must be forwarded to the Chairperson by close of business on the Friday before the next scheduled meeting. The agenda with attached meeting papers will be distributed on the Monday before to the next scheduled meeting.

#### 3.8 Notes and Meeting Papers

The notes of each Consumer and Community Reference Group meeting will be prepared by Ms Helen Daley, Administrative Secretary. Full copies of the notes, including attachments, shall be provided to all members no later than the Friday following each meeting. By agreement with the Group, out-of-session decisions will be deemed acceptable. Where agreed, all out-of-session decisions shall be recorded in the notes of the next scheduled Consumer and Community Reference Group.

### 4. Terms of Reference

To provide the Project Steering Committee with comment and advice on:

• The management of data, publications, and the protection of individual and/or community identities during the project;

• The methodology, conduct, dissemination of results and potential outcomes of the project;

• Alcohol use in pregnancy and how it is dealt with in a culturally sensitive way;

• Project documents such as consent forms and participant information sheets, interview guides, questionnaires and health promotion resources;

• The final report and draft reports and manuscripts before they are published in scientific journals, and

• The preparation and development of summaries for the community and dissemination.

### 5. Values Underpinning the Terms of Reference

The Consumer and Community Reference Group Terms of Reference are underpinned by the *National Health and Medical Research Council. A Model Framework for Consumer and Community Participation in Health and Medical Research, Canberra: Australian Government; 2005*.

## Appendix 2: Alcohol and Pregnancy Project: Information for the Community

Fetal Alcohol Spectrum Disorder (FASD) describes a range of effects that can occur in an individual whose mother drank alcohol during pregnancy. The effects may include physical, mental, behavioural, and/or learning disabilities with possible lifelong effects. Fetal Alcohol Syndrome (FAS) represents the severe end of the range of abnormalities resulting from alcohol use in pregnancy.

Western Australian (WA) data show that Aboriginal children are over 100 times more likely to be diagnosed with FAS. Alcohol use in pregnancy and its effects, and FAS in particular, have received limited attention in Australia.

The *Alcohol and Pregnancy Project *is funded by Healthway for three years and builds on our previous research where we identified that health professionals' lack knowledge about the diagnosis of FAS. Most health professionals do not enquire about or provide women with information on the consequences of alcohol use during pregnancy, and most reported their need for educational resources such as written material for themselves and for distribution to clients.

The objectives of the *Alcohol and Pregnancy Project *are:

1. To provide health professionals in WA with educational resources for advising women on alcohol use during pregnancy and its effects;

2. To provide health professionals in WA with educational resources that can be given out to women to supplement advice on alcohol use in pregnancy and its effects; and

3. To increase the proportion of health professionals in WA who:

a. routinely ask pregnant women about alcohol use; and

b. routinely provide women with information about the consequences of drinking alcohol during pregnancy.

Consultation and participation with consumers and the community is an important part of this project and Reference Groups will be set up with agreed terms of reference. In addition, focus groups will also be held with women in the community to guide the development of the educational resources.

Some of the expected benefits from this project include:

1. Providing communities and individuals with an accessible resource that is not currently available;

2. Providing links to a 24-hour information and clinical advisory service;

3. Providing a register of specialists who can be contacted about the effects of alcohol in pregnancy;

4. Producing results which will benefit Australian policy makers by providing them with the evidence with which to review policies and guidelines about alcohol use in pregnancy and its effects.

Dissemination of research findings is a major priority of this project. Summaries for the community, newsletters, and articles for journals will be produced and made available to the public via a website. The findings will be distributed to government and non-government agencies to inform health policy and practice.

For further information about the *Alcohol and Pregnancy Project*, or for a copy of the paper from our previous research, please contact: Ms Jan Payne, Telethon Institute for Child Health Research, phone (08) 9489 7777, email janp@ichr.uwa.edu.au

## Appendix 3: Alcohol and Pregnancy Project: Project Summary

### Background

Fetal Alcohol Spectrum Disorder (FASD) describes a range of effects that can occur in an individual whose mother drank alcohol during pregnancy. The effects may include physical, mental, behavioural, and/or learning disabilities with possible lifelong implications. Fetal Alcohol Syndrome (FAS) represents the severe end of the spectrum of abnormalities resulting from alcohol exposure in pregnancy. Western Australian (WA) data show that Aboriginal children are over 100 times more likely to be diagnosed with FAS. Alcohol use in pregnancy and its effects, and FAS in particular, have received limited attention in Australia. Many health professionals are not well prepared to provide guidance to women about alcohol use and its effects in pregnancy and FAS.

This proposal builds our previous research *Fetal Alcohol Syndrome in Australia *(Healthway project 10563; WA Aboriginal Health Information Ethics Committee reference 66-3/02; Princess Margaret Hospital registration 533/EP and 676/EP) where we conducted Australia-wide surveillance of FAS, and surveys of WA health professionals' knowledge, beliefs and practices in relation to alcohol use in pregnancy and FAS. The health professionals included Aboriginal health workers, allied health professionals, community nurses, general practitioners, obstetricians and paediatricians. We identified that health professionals lack knowledge about diagnosis and management of FAS, and about alcohol use during pregnancy, and the National Health and Medical Research Council (NHMRC) guidelines regarding alcohol use in pregnancy. Health professionals reported their need for educational resources such as written material for themselves and for distribution to clients, checklists (FAS diagnostic criteria and alcohol use in pregnancy), training, and a registry of specialists and referral resources.

### Objectives

The objectives of this research are:

1. To provide health professionals in WA with educational resources for advising women on alcohol use during pregnancy and its effects;

2. To provide health professionals in WA with educational resources that can be given out to women to supplement advice on alcohol use in pregnancy and its effects; and

3. To increase the proportion of health professionals in WA who routinely ask pregnant women about alcohol use, and who routinely provide women with information about the consequences of drinking alcohol during pregnancy.

### Research Plan

This research will be conducted from 2006-2008 and is funded by Healthway. Health professionals in WA will be provided with educational resources for advising women and for giving to women to supplement advice on alcohol use during pregnancy and its effects on the fetus and child. To develop the resources we will synthesise international and Australian published material and collaborate with experts in the field (WA Aboriginal Community Controlled Health Organisations, professional colleges, state and federal health organisations). We will conduct focus groups and in-depth interviews in metropolitan and regional areas of WA with Aboriginal and non-Aboriginal community members and health professionals to explore issues relating to the communication of information on alcohol use in pregnancy and its effects on the fetus and child. An Aboriginal person will be trained in focus group facilitation and will conduct focus groups for Aboriginal community members and health professionals. The educational resources will be developed, pre-tested and amended before distribution to health professionals throughout WA. We anticipate the educational resources will include information on preventing Fetal Alcohol Syndrome, screening for alcohol use in pregnancy, diagnosing and treating Fetal Alcohol Syndrome, and imparting information without causing guilt or stigma to pregnant women and those of childbearing age. We will also provide links to a 24-hour information and clinical advisory service and establish a register of specialists (who can be contacted about the effects of alcohol use in pregnancy) for use by health professionals and the community. After distribution of the educational resources we will conduct a survey of health professionals using similar methods to the recent research titled *Fetal Alcohol Syndrome in Australia. *We will evaluate whether there has been an increase in the proportion of health professionals who routinely ask and provide information to pregnant women about the consequences of drinking alcohol in pregnancy. We will also evaluate the public health impact of the effectiveness of the research.

### Risks and benefits of the research

1. This research undertakes to do no harm to participants or contributors and no unintended consequences are expected to arise from or after this research. An information sheet outlining the topic of alcohol in pregnancy will have been read by participants of focus groups and in-depth interviews (and informed consent obtained) but it is possible that the topic of alcohol use in pregnancy may cause anxiety or raise concern in some participants. The investigators will ensure that appropriate services such as counselling are available for participants (should the need arise) before the focus groups and in-depth interviews are commenced.

2. Community members who volunteer to participate in focus groups in metropolitan and regional areas of WA will benefit from knowing they have made a valuable contribution to research aimed at preventing harm to individuals who were exposed to alcohol during pregnancy.

3. Health professionals will benefit from being provided with educational resources that will increase their knowledge and enable them to implement health promotion aimed at reducing alcohol use in pregnancy and its effects on the fetus and child. Health professionals will also be better informed about the effects of alcohol use in pregnancy and diagnosing and managing children with Fetal Alcohol Syndrome and individuals who have been affected by exposure to alcohol in pregnancy.

4. The information developed in this research will benefit communities and individuals in communities by providing an accessible resource and links that are not currently available to a 24-hour information and clinical advisory service and a register of specialists who can be contacted about the effects of alcohol in pregnancy.

5. The results of the research will benefit Australian policy makers by providing them with the evidence with which to review policies and guidelines about alcohol use in pregnancy and its effects.

6. Health professionals will be better prepared to advise women about the affects of alcohol use in pregnancy on the fetus and child and this will benefit women by enabling them to make informed choices about alcohol use in pregnancy.

7. Fewer children will be born affected by alcohol exposure during pregnancy, and who along with their families and the community, will be spared the lifelong consequences of this exposure.

### Ethics

1. The investigators involved in this research are committed to consultation and communication, the importance of cultural sensitivity, and respect and recognition of deeply held values of Aboriginal and Torres Strait Islander peoples. The investigators have seriously considered the six values (spirit and integrity, reciprocity, respect, equality, responsibility, and survival and protection) in Aboriginal and Torres Strait Islander health research that guide investigators in the conception, design, and conduct of research involving Aboriginal and Torres Strait Islander peoples.

2. Ethical approval for this study has been requested from the WA Aboriginal Health Information Ethics Committee and the Princess Margaret Hospital Ethics Committee. WA Community Controlled Health Organisations throughout WA will be consulted. Participants in focus groups and in-depth interviews will give informed consent and return of the questionnaire will indicate consent for participants in the survey of health professionals.

3. Information given by participants of focus groups, in-depth interviews and the survey of health professionals will not be named and individuals will not be identified. Individual participants will not be identified in any publications or reports arising from the project.

4. All data will be maintained in strict confidence, securely locked in metal cabinets when not in use. Investigators computers will be password and firewall protected.

### Dissemination of results

The dissemination of research findings and outcomes is a major priority of this project. Publications in peer reviewed journals and newsletters will disseminate the findings to health professionals, along with presentations at conferences, both nationally and locally. The results will be distributed to government and non-government health professional bodies to inform health policy and practice.

### Chief investigators

Clinical Professor Carol Bower, Telethon Institute for Child Health Research, Perth, WA

Associate Professor Elizabeth Elliott, Australian Paediatric Surveillance Unit, Sydney, NSW

Associate Professor Nadine Henley, Edith Cowan University, Perth, WA

Ms Jan Payne, Telethon Institute for Child Health Research, Perth, WA

Mrs Colleen O'Leary, Telethon Institute for Child Health Research, Perth, WA

Ms Heather D'Antoine, Telethon Institute for Child Health Research, Perth, WA

Associate Professor Anne Bartu, Department of Health, Perth, WA

### Associate investigators

Ms Lynda Blum, Department of Health, Perth, WA

Ms Roslyn Giglia, Curtin University of Technology, Perth, WA

Dr Janet Hammill, University of Queensland, Queensland

Associate Professor Ray James, Curtin University of Technology, Perth, WA

Dr Christine Jeffries-Stokes, Rural Peadiatric School, Kalgoorlie, WA

Dr Anne Mahony, Department of Health, Broome, WA

Mr Daniel McAullay, Department of Health, WA

Ms Anne McKenzie, Telethon Institute for Child Health Research, Perth, WA

### Project team

Ms Melinda Berinson

Ms Kathryn France

Professor Nadine Henley

Ms Heather Monteiro

Ms Jan Payne

### Reference

1. J Payne, E Elliott; H D'Antoine, E Haan, C Bower: **Health professionals' knowledge, practice and opinions about fetal alcohol syndrome and alcohol consumption in pregnancy**. *Aust N Z J Public Health *2005, **29**(6):558-564.

### Contact person

Project Manager: Ms Jan Payne

The Telethon Institute for Child Health Research

Population Sciences

Mailing Address PO Box 855, WEST PERTH WA 6872

Telephone: 08 9489 7752

Fax: 08 9489 7700

Email: janp@ichr.uwa.edu.au
